# Insights into substrate coordination and glycosyl transfer of poplar cellulose synthase-8

**DOI:** 10.1016/j.str.2023.07.010

**Published:** 2023-08-11

**Authors:** Preeti Verma, Albert L. Kwansa, Ruoya Ho, Yaroslava G. Yingling, Jochen Zimmer

**Affiliations:** 1 Department of Molecular Physiology and Biological Physics, University of Virginia, Charlottesville, VA 22903, USA; 2 Department of Materials Science and Engineering, North Carolina State University, Raleigh, NC 27695, USA; 3 Howard Hughes Medical Institute, University of Virginia, Charlottesville, VA 22903, USA; 4 Lead contact

## Abstract

Cellulose is an abundant cell wall component of land plants. It is synthesized from UDP-activated glucose molecules by cellulose synthase, a membrane-integrated processive glycosyltransferase. Cellulose synthase couples the elongation of the cellulose polymer with its translocation across the plasma membrane. Here, we present substrate- and product-bound cryogenic electron microscopy structures of the homotrimeric cellulose synthase isoform-8 (CesA8) from hybrid aspen (poplar). UDP-glucose binds to a conserved catalytic pocket adjacent to the entrance to a transmembrane channel. The substrate’s glucosyl unit is coordinated by conserved residues of the glycosyltransferase domain and amphipathic interface helices. Site-directed mutagenesis of a conserved gating loop capping the active site reveals its critical function for catalytic activity. Molecular dynamics simulations reveal prolonged interactions of the gating loop with the substrate molecule, particularly across its central conserved region. These transient interactions likely facilitate the proper positioning of the substrate molecule for glycosyl transfer and cellulose translocation.

## In brief

Verma et al. determined the cryo-electron microscopy structure of UDP-glucose-bound poplar cellulose synthase-8 that provides insights into substrate selectivity and glycosyl transfer. Site-directed mutagenesis of a conserved but structurally flexible gating loop reveals residues critical for cellulose biosynthesis. Molecular dynamics simulations of substrate-bound cellulose synthase-8 reveal direct gating loop-substrate interactions.

## Graphical Abstract

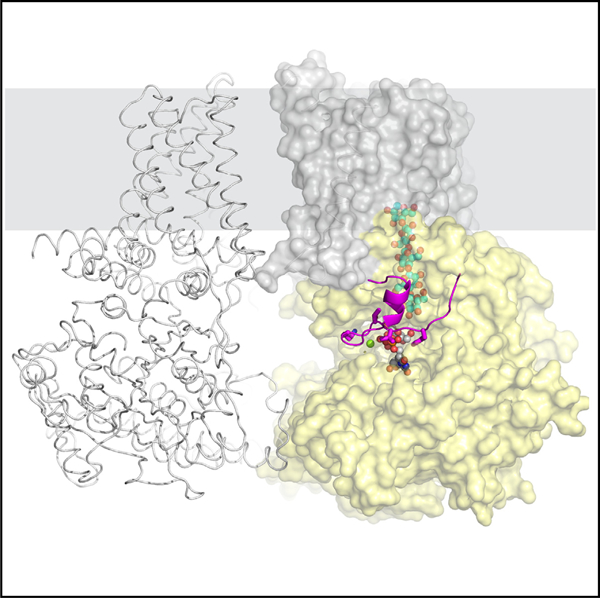

## INTRODUCTION

Cellulose is an abundant biopolymer that is produced primarily by land plants as a structural cell wall component. Because plants produce cellulose from photosynthetically synthesized glucose molecules, the polysaccharide is a major atmospheric carbon dioxide sink as well as a significant renewable energy resource.^1^

Cellulose’s glucosyl units are connected via β-(1,4)-glycosidic linkages that enable an approximately 180° rotation of neighboring sugar units within the polymer.^2^ The resulting amphipathic polysaccharide can be organized into cable-like fibrillar structures, so-called cellulose micro- and macrofibrils, that are spun around the cell as a load-bearing wall component.^3^ Cellulose is synthesized from UDP-activated glucose (UDP-Glc) by cellulose synthase (CesA), a membrane-integrated processive family-2 glycosyltransferase (GT).^4,5^

CesA catalyzes glucosyl transfer from UDP-Glc (the donor sugar) to the C4 hydroxyl group at the non-reducing end of the nascent cellulose polymer (the acceptor). Following chain elongation, CesA also facilitates cellulose translocation across the plasma membrane through a pore formed by its own transmembrane (TM) segment. To couple cellulose synthesis with secretion, CesA’s catalytic GT domain packs against a channel-forming TM region via three conserved amphipathic interface helices (IF1–3).^6,7^

Cellulose biosynthesis is evolutionarily conserved, with homologous pathways found in prokaryotes, oomycetes, and some animals. Previous work on bacterial cellulose biosynthetic systems from *Gluconacetobacter xylinus* (formerly *Acetobacter xylinum*),^8,9^ *Rhodobacter sphaeroides*,^10^ and *Escherichia coli* (*E. coli*)^11^ provided detailed insights into the reaction mechanism and enzyme regulation,^12–15^ as well as cellulose secretion,^7^ assembly,^16,17^ and modification.^18^ Further, recent cryogenic electron microscopy (cryo-EM) studies on trimeric plant CesA complexes confirmed an evolutionarily conserved enzyme architecture, in support of an equally conserved catalytic reaction mechanism.^19,20^

To delineate principles underlying substrate selectivity and catalysis, we determined cryo-EM structures of the full-length poplar CesA isoform-8 (CesA8) bound to either UDP-Glc or the product and competitive inhibitor UDP. The obtained complexes demonstrate substrate binding to a conserved pocket at the interface between the enzyme’s cytosolic catalytic domain and TM region. However, in contrast to the bacterial homolog BcsA where a conserved ‘‘gating loop’’ stabilizes UDP-Glc at the active site,^7^ this loop is disordered in the substrate-bound CesA8 complex. Extensive mutagenesis analyses of the loop’s conserved FxVTxK motif in *Rhodobacter sphaeroides* BcsA as well as poplar CesA8 underscore its importance for catalytic activity. All-atom molecular dynamics simulations indeed confirm prolonged interactions of the loop with the substrate molecule and suggest its role in proper positioning of the substrate for glycosyl transfer.

## RESULTS

### Cryo-EM analyses of nucleotide-bound poplar CesA8

Poplar CesA8 was expressed in Sf9 insect cells and purified in the detergents lauryl maltose neopentyl glycol/cholesteryl hemisuccinate and glyco-diosgenin as previously described^19^ and summarized in the STAR methods. Under these conditions, the enzyme is catalytically active, synthesizing cellulose *in vitro* in the presence of UDP-Glc and magnesium ions. Alongside cellulose, CesA generates UDP as a second reaction product of the glycosyl transfer reaction. Previous studies on bacterial and plant cellulose synthases as well as other related family-2 GTs demonstrated that UDP competitively inhibits the enzymes, due to its interactions with the catalytic pocket.^10,21,22^ This observation has been exploited to obtain UDP-inhibited structures of hyaluronan and cellulose synthases.^14,23^

**Figure 1.**
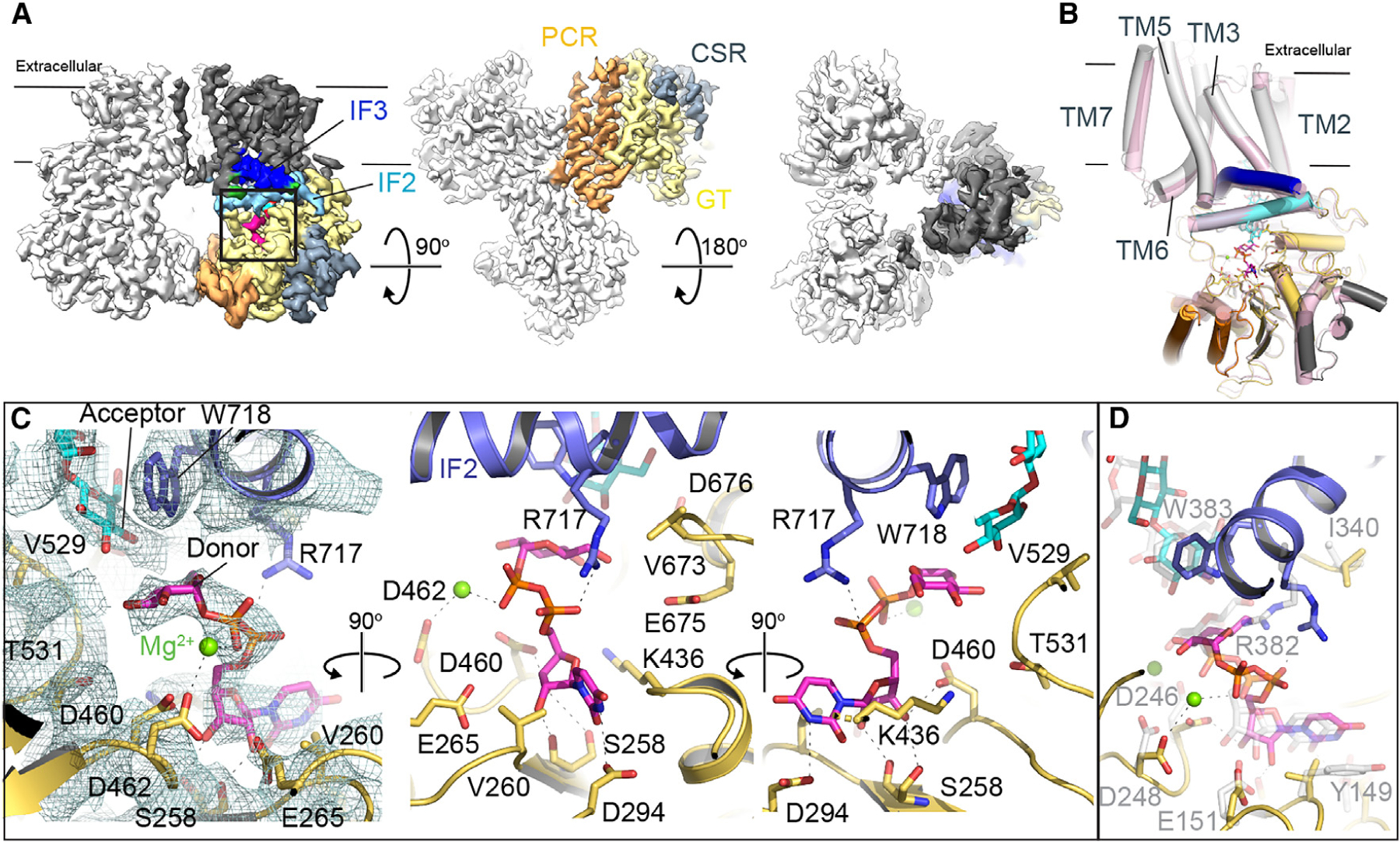
Structure of UDP-glucose-bound poplar CesA8 (A) Cryo-EM map of the substrate-bound poplar CesA8 trimer. Two subunits are shown in light gray and one subunit is colored according to its domains: dark gray: TM region, wheat: GT domain, orange: PCR, steel blue: CSR, and light blue and blue for IF helices 2 and 3, respectively. See also Figures S1, S2, and S3A. (B) Overlay of one subunit of the apo (PDB: 6WLB, colored pink) and substrate (UDP-Glc)-bound CesA8 structures. The substrate-bound CesA8 structure is colored as in panel (A). (C) Zoom-in view of the active site and substrate coordination. The cryo-EM map of the substrate and surrounding residues is shown as a mesh in the left panel. (D) Superimposition of substrate-bound BcsA (PDB: 5EIY) and poplar CesA8. BcsA is colored light gray for its carbon atoms. See also Figure S3.

We determined UDP and UDP-Glc-bound poplar CesA8 structures by incubating the purified enzyme with 5 mM of nucleotide and 20 mM MgCl_2_ prior to cryo grid preparation (see STAR methods, Figure S1). The ligand-bound CesA complexes were imaged and processed as described before^19^ (Figures S1 and S2; Table S1). Overall, the trimeric organization of CesA8 is preserved in UDP and UDP-Glc-bound states, suggesting that the complex indeed represents a biologically functional unit (Figure 1A). Within the resolution limits of our cryo-EM maps (approximately 3.5 Å), the UDP moiety adopts the same binding pose in the UDP-only and UDP-Glc-bound states (Figure S3A); hence, the following discussion focuses on the substrate-bound conformation. The UDP-Glc-bound structure is very similar to the previously solved apo CesA8 structure,^19^ with a root-mean-square deviation between Cα atoms of 0.8 Å (Figure 1B). Each CesA8 protomer also contains a nascent cellulose polymer within the TM channel. The polymer’s first five glucosyl units, starting at the non-reducing end near the catalytic pocket, are sufficiently well ordered to allow modeling (Figure S2). As described previously,^19^ the terminal acceptor glucosyl unit rests next to Trp718 of the conserved QxxRW motif, right above the substrate binding pocket (Figure 1C).

### CesA8 positions the donor sugar beneath the acceptor glucosyl unit

CesA8’s GT domain forms a classical GT-A fold with a central mixed β-sheet surrounded by α-helices.^24^ The bound substrate molecule is coordinated by conserved residues distributed throughout the GT domain^6^ (Figure 1C). First, the substrate’s uridine group is sandwiched between Glu265 and Lys436 and fits into a groove created by Ser258 and Val260 of the conserved STVDP motif belonging to the first β-strand of the GT-A fold. Second, Asp294 of the invariant DDG motif terminating β-strand #2 is in hydrogen bond distance to the Nε ring nitrogen of the uracil moiety. Third, the conserved DxD motif (Asp460 and Asp462), following β-strand #5, contributes to the coordination of a magnesium cation, which is also in contact with the substrate’s β-phosphate. Additionally, the nucleotide’s diphosphate group interacts with Arg717 of the QxxRW motif originating from IF-2 (Figure 1C).

The donor sugar fits into a polar pocket directly underneath the acceptor glucosyl unit of the nascent cellulose chain. This pocket is proximal to the water-membrane interface formed from IF-2, the finger helix that is N-terminally capped with the invariant VTED motif (residues 673 to 676), as well as the backbone of Val529, Gly530, and Thr531 belonging to the conserved YVGTG motif (Figure 1C). Potential hydrogen bond donors and acceptors from protein side chain and back-bone regions surround the donor glucosyl unit. However, all observed distances to the donor’s hydroxyl groups exceed 3.5 Å, suggesting that the substrate molecule is not fully inserted into the catalytic pocket. Accordingly, the distance between the acceptor’s C4 hydroxyl and the donor’s C1 carbon exceeds 5.5 Å (Figure 1C). The observed substrate coordination is consistent with interactions delineated for bacterial BcsA bound to a non-hydrolysable UDP-Glc phosphonate analog^7^ (Figure 1D), as well as UDP-N-acetylglucosamine bound to chitin and hyaluronan synthases^23,25,26^ (Figures S3B–S3D).

### The flexible gating loop is required for catalytic activity

**Figure 2.**
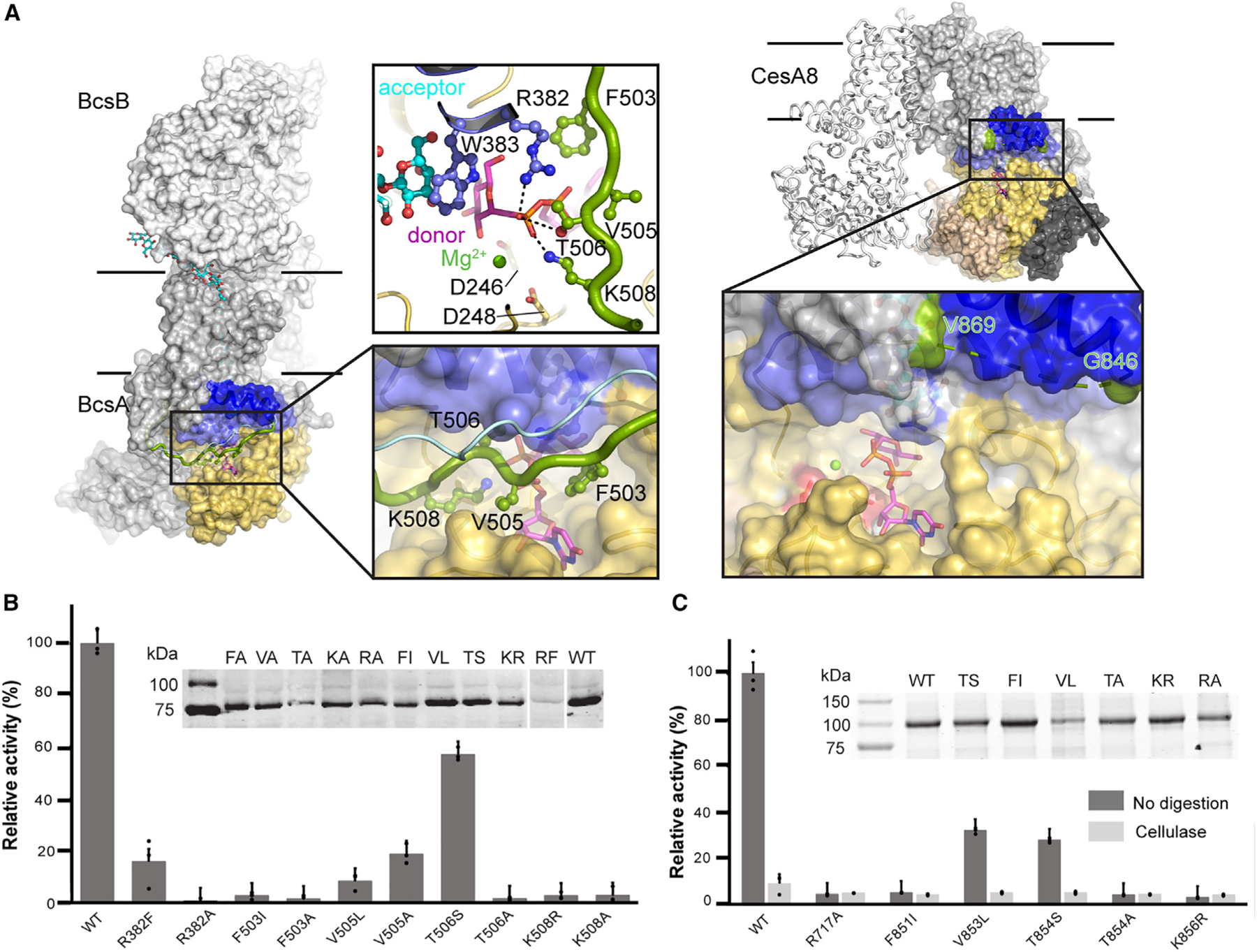
The gating loop is critical for catalytic activity (A) Substrate-bound structures of bacterial (BcsA, PDB: 5EIY) and poplar cellulose synthase (this study). The substrate is shown as sticks colored magenta for carbon atoms. The gating loop positions observed in BcsA in the absence (BcsA, PDB: 4P02) and the presence of substrate are shown as light blue and green ribbons, respectively. (B) Comparison of the catalytic activities of the bacterial BcsA-B wild-type (WT) complex and its variants containing the indicated BcsA mutants. Experiments were performed in IMVs and activities were measured upon incorporation of tritium-labeled glucose into cellulose. The product yield for the WT enzyme was set to 100%. Inset: Western blot of the BcsA-B containing IMVs used for activity assays detecting His-tagged BcsA. Blotting was done to normalize the concentration of mutants with respect to the WT enzyme. The lanes for the ‘‘RF’’ and ‘‘WT’’ constructs were moved from the left to the right side of the Western blot for clarity. (C) Catalytic activity determined for WT CesA8 and its mutants. Reactions were performed in the presence or absence of cellulase using affinity-purified proteins. The product obtained for the undigested WT CesA8 was set as 100%. Inset: Coomassie stained 10% SDS-PAGE of the purified CesA8 constructs used for normalization and activity assays. In panels (B) and (C) error bars represent positive standard deviations from means of three replicas. See also Figure S4.

CesA8’s IF3 is connected to TM helix 5 via a ~20 residue long cytosolic gating loop that runs roughly across the opening of the catalytic pocket. The gating loop contains a conserved FxVTxK motif but is not resolved in all cryo-EM maps of CesAs, most likely due to conformational flexibility (Figure 2A).

Crystallographic analyses of *Rhodobacter sphaeroides* BcsA revealed different conformations of the gating loop.^6,14^ The loop retracts from the catalytic pocket in a nucleotide-free state and inserts into it in the presence of either UDP or a substrate analog. In the inserted state, the conserved FxVTxK motif contacts the UDP moiety, thereby likely stabilizing it at the active site. In addition to substrate stabilization, gating loop insertion into the catalytic pocket also facilitates translocation of the nascent polysaccharide between elongation steps.^7^ Unlike for BcsA, nucleotide binding to CesA8 does not stabilize the gating loop in a similar manner (Figure 2A).

To probe the functional significance of the gating loop’s FxVTxK motif, we performed site-directed mutagenesis and *in vitro* functional analyses of the bacterial BcsA and poplar CesA8 enzymes. For BcsA, replacing Phe503 of the FxVTxK motif with Ala or the bulky hydrophobic residue Ile abolishes catalytic activity (Figures 2B and S4). Substituting the conserved Val505 residue with Ala or Leu dramatically reduces catalytic activity to about 20% and 10%, respectively, relative to the wild-type enzyme. A drastic reduction is observed when the following Thr506 residue is replaced with Ala, yet its substitution with Ser retains about 60% relative catalytic activity. The Lys508 residue at the C terminus of the FxVTxK motif is also critical for function; neither an Ala nor an Arg residue at this position supports enzymatic activity (Figure 2B).

Upon insertion into the catalytic pocket, Phe503 of BcsA’s gating loop forms cation-π interactions with Arg382 of the conserved QxxRW motif located in IF2 at the cytosolic water-lipid interface.^14^ In this position, Arg382 forms a salt bridge with the substrate’s diphosphate group, as also observed in CesA8 (Figures 1D and 2A). This residue is critical for catalytic activity as its substitution with Ala renders BcsA inactive, while its substitution with Phe retains about 16% activity (Figure 2B).

A similar analysis was performed of CesA8’s FxVTxK motif. Here, we replaced Phe851 with Ile, V853 with Leu, Thr854 with Ser or Ala, and Lys856 with Arg. Due to increased background readings in membrane vesicles, the mutant enzymes were purified and analyzed for catalytic activity in a micelle-solubilized state, as previously described.^19^ Of the generated mutants, only the V853L and T854S substitutions retain about 30% catalytic activity, relative to the wild-type enzyme. Further, as observed for BcsA, replacing Arg717 of CesA8’s QxxRW motif with Ala renders the enzyme catalytically inactive (Figure 2C).

### Molecular dynamics simulations reveal persistent gating loop-substrate interactions

**Figure 3.**
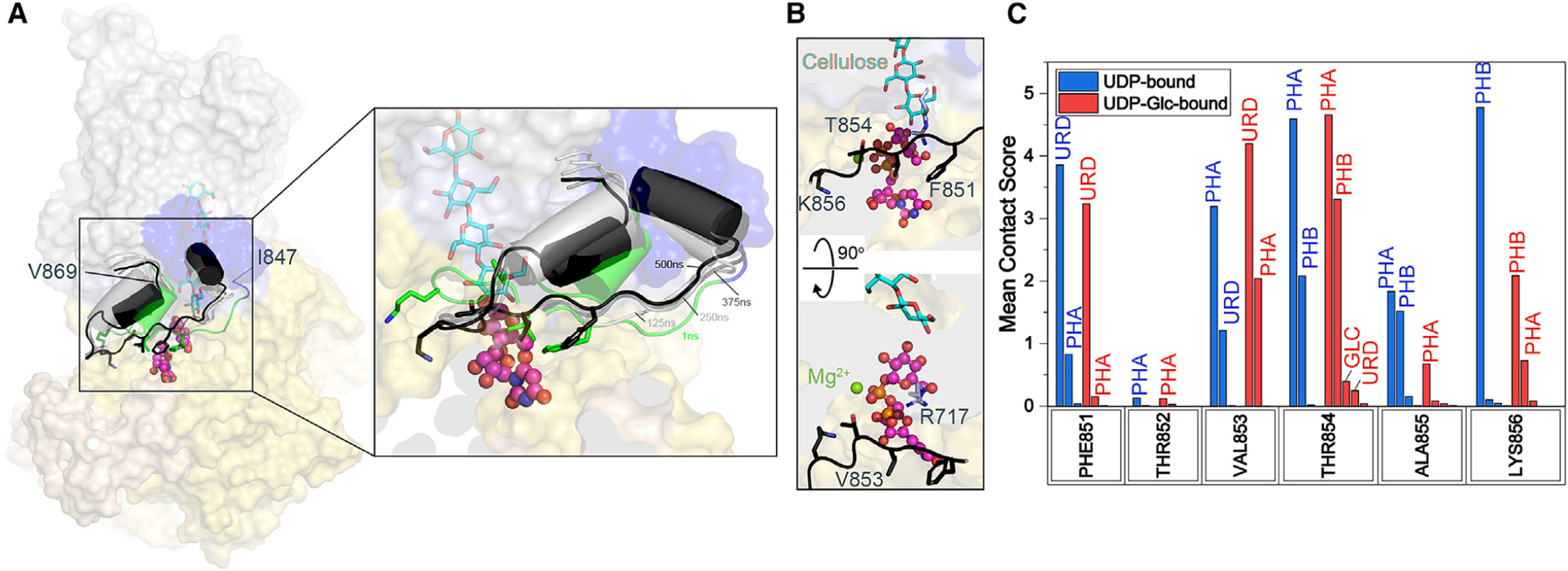
Molecular dynamics simulation analysis of gating loop-UDP interactions (A) Overlay of UDP-Glc-bound CesA8 models with an inserted gating loop after 1, 125, 250, 375, and 500 ns of MD simulation. The original model is shown as a surface-colored structure as in Figure 1. The gating loop is shown as a cartoon for models obtained after the indicated simulation times. Right panel shows magnified view of the boxed area. (B) Close-up view of interactions between the gating loop and the substrate’s uracil moiety after 500 ns of MD simulation. (C) A bar plot of mean contact scores for individual CesA gating loop residues interacting with UDP/UDP-Glc and/or Mg^2+^. Mean contact score refers to a mean over 1,000 frames over time (500 ns), and each bar represents an average from three independent simulations. URD: Uridine, PHA/B: alpha and beta phosphate, GLC: alpha-D-glucosyl group. Generated with Origin.^30^ See also Figure S5.

To probe the interactions between CesA8’s gating loop and a nucleotide at the active site, we examined the dynamics of the gating loop in the presence of UDP and UDP-Glc. The initial conformation of the loop was modeled based on the loop’s position in the substrate-bound BcsA crystal structure (PDB: 5EIY) as well as weak discontinuous gating loop density observed at low contour levels in the CesA8 cryo-EM map (Figures 3 and S5). All-atom molecular dynamics simulations of UDP- and UDP-Glc-bound CesA8 monomers with a phospholipid bilayer, water, and NaCl were performed for 500 ns, in triplicate, as detailed in the STAR methods. Relative to its starting conformation, the gating loop remains in close contact with the nucleotide during the simulations, with greatest fluctuations observed for regions N and C terminal to the conserved FxVTxK motif (Figure 3A). For the UDP-bound case, notable contacts are observed for all residues of the FxVTxK motif, except for Thr852. For the UDP-Glc-bound case, a similar set of residues of the FxVTxK motif are found to have influential interactions. In both cases, residues Phe851, Val853, Thr854, and Lys856 are predicted to interact the most with UDP or UDP-Glc, associated with relatively high mean contact scores. Phe851 and Val853 are positioned to surround the substrate’s uracil group, while Thr854 interacts with the ligand’s alpha phosphate (Figure 3B). Furthermore, the C-terminal Lys856 of the FxVTxK motif is predicted to interact primarily with the ligand’s beta phosphate. The mean contact score metric employed in PyContact provides a measure of contact persistence over time, contact proximity, and contact area. Thus, mean contact scores associated with the gating loop residues and UDP/UDP-Glc/Mg^2+^ reveal relatively high contact persistence, proximity, and/or area, especially for the four conserved residues of the FxVTxK motif (Figure 3C; Tables S2 and S3).

## DISCUSSION

CesA catalyzes multiple reactions: First, the formation of a linear β-(1,4)-glucan; second, the secretion of the polysaccharide into the extracellular milieu; and third, due to the self-assembly of CesAs into supramolecular complexes, the coalescence of cellulose polymers into fibrillar structures.^4,5^

Polymer secretion is achieved by closely associating the catalytic cytosolic GT domain with a channel-forming TM segment, with residues from both the regions contributing to donor and acceptor coordination.

**Figure 4.**
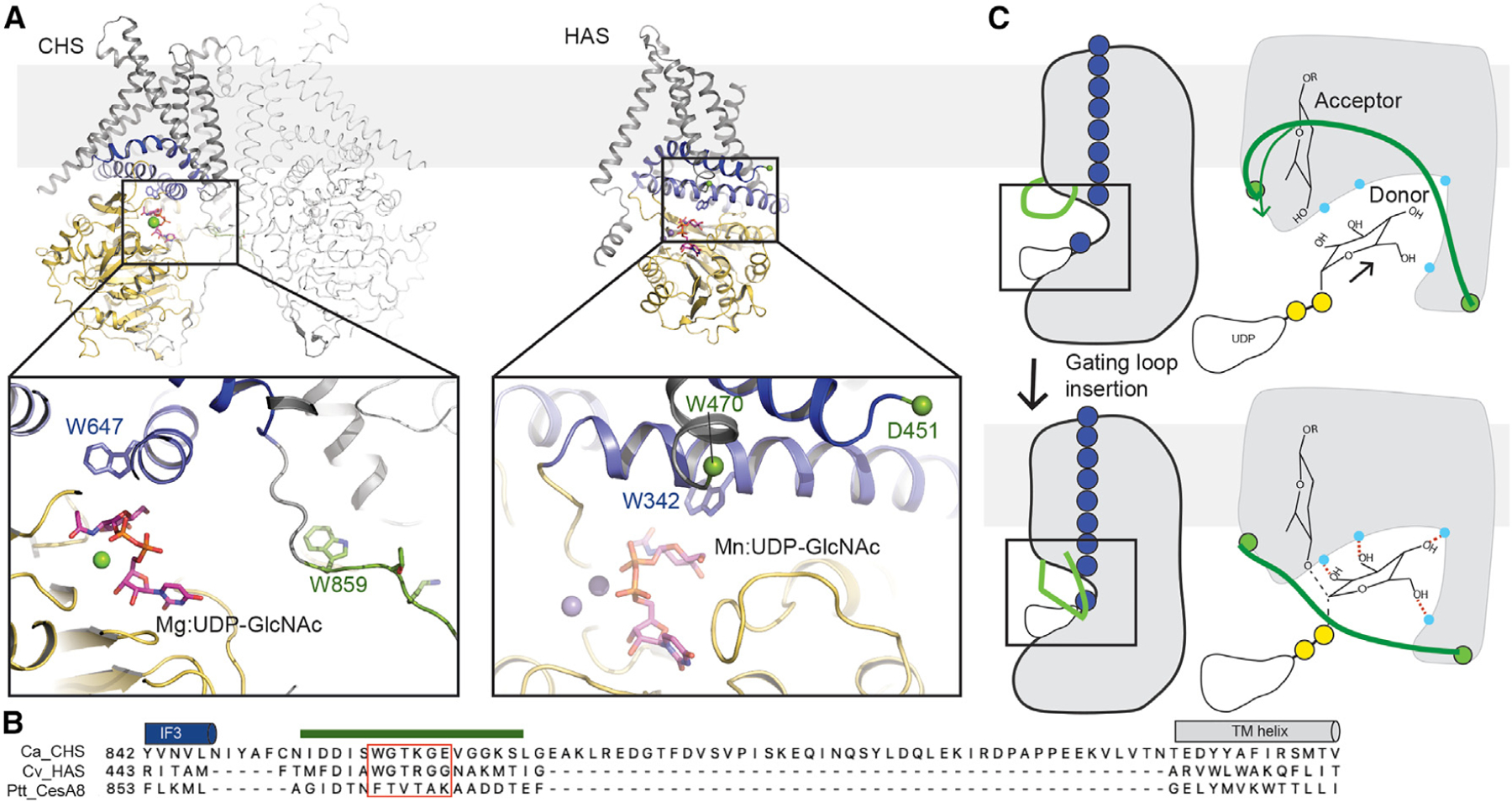
Localization and proposed function of the gating loop in membrane-integrated processive GT-2 glycosyltransferases (A) Substrate-bound structures of *Candida albicans* chitin synthase (CHS, PDB: 7STM) and Chlorella virus hyaluronan synthase (HAS, PDB: 7SP8). Both enzymes place a conserved WGTR/KG motif near the catalytic pocket (unresolved in HAS). (B) Alignment of the gating loop regions of chitin, hyaluronan, and cellulose synthases. (C) Gating loop insertion likely positions the substrate molecule closer to the base catalyst and helps in accepting glycosyl unit. Left panel: Side view of entire enzyme, right panel: Close-up view of the active site. Conformational changes of the donor sugar may increase the reactivity of the electrophile.

The observed binding pose of the substrate UDP-Glc at CesA8’s active site is consistent with other structures of processive and non-processive GT-A enzymes, including hyaluronan and chitin synthases^23,25,26^ (Figures 4A and 4B). CesA coordinates UDP with invariant sequence motifs, primarily localized at the edge of its central GT-A β-sheet. The donor sugar, attached to UDP, is positioned in a hydrophilic pocket near the water-lipid interface. This pocket is created by the GT domain as well as amphipathic interface helices that establish the transition from the cytosolic to the TM region. The observed substrate coordination contrasts a recently reported UDP-Glc-bound crystal structure of the GT core of *Arabidopsis* CesA3,^27^ which exhibits an unphysiological catalytic pocket.

Compared to UDP or substrate-bound states of bacterial BcsA, CesA8’s conserved gating loop remains disordered in the substrate-bound cryo-EM structure. Weak map density ‘‘above’’ the entrance to the catalytic pocket (Figure S5A) indicates flexibility of the gating loop, as also observed for BcsA in nucleotide-free states.^14^ Site-directed mutagenesis of the gating loop, however, demonstrates its profound importance for cellulose biosynthesis. We hypothesize that the gating loop inserts transiently into the catalytic pocket to position the substrate for glycosyl transfer and perhaps decreasing the distance between the donor and acceptor glucosyl units (Figure 4C). Substrate repositioning may also (a) favor protein interactions with the donor’s hydroxyl groups to foster substrate specificity, and (b) facilitate conformational changes of the donor’s pyranose ring to increase the reactivity of its C1 carbon.

Hyaluronan and chitin synthases also contain putative gating loops. The corresponding sequence is WGTR/KG instead of FxVTxK (Figure 4B). This motif is located in a loop following IF3 near the active site. It is disordered in hyaluronan synthase structures or bridges a dimerization motif in chitin synthase (Figure 4A). Substrate-bound structures of these enzymes also suggest incomplete insertions of the substrate molecules into the catalytic pockets, based on the assumed acceptor binding site formed by the Trp of the conserved QxxRW motif. As for CesA, transient gating loop insertion could enforce a substrate conformation favorable for glycosyl transfer (Figure 4C).

Cellulose microfibrils are synthesized from CesA complexes (CSC) resembling 6-fold symmetric particles.^28^ The repeat unit is likely represented by the trimeric cryo-EM structures obtained for poplar and cotton CesAs.^19,29^ Within a CSC, multiple CesAs synthesize and secrete cellulose polymers to facilitate their alignment into a microfibril. It is currently unknown whether the individual CesA activities are coordinated. Our substrate-bound cryo-EM structures do not suggest allosteric regulation of the catalytic activities within a CesA trimer. However, we cannot exclude interprotomer crosstalk in the context of a fully assembled CSC, perhaps mediated by currently unresolved regions.

## STAR★METHODS

### RESOURCE AVAILABILITY

#### Lead contact

Further information and request for resources and reagents should be directed to and will be fulfilled by the lead contact, Jochen Zimmer (jz3x@virginia.edu).

#### Materials availability

This study did not generate new unique reagents. Plasmids generated in this study will be available upon request to the lead contact.

#### Data and code availability

- The coordinates of UDP-bound CesA8 and UDP-Glucose bound CesA8 have been deposited to the Protein Data Bank under accession numbers 8G27 and 8G2J. CryoEM maps of the UDP-bound CesA8 and UDP-Glucose bound CesA8 have been deposited in the Electron Microscopy Data Bank under the accession numbers 29678 and 29679 respectively. All the deposited data are publicly available as of the date of publication and accession numbers are also listed in the key resources table.
- This paper analyzes existing, publicly available data. These accession numbers for the datasets are listed in the key resources table.
- This paper does not report original code data.
- Any additional information required to reanalyze the data reported in this paper is available from the lead contact upon request.

### EXPERIMENTAL MODEL AND STUDY PARTICIPANT DETAILS

#### Bacterial strains

*E*. *coli* Rosetta 2 (DE3) cells (Novagen) were used in this study for recombinant protein production. Cells were cultured in ZYP-5052 auto-induction media^42^ supplemented with necessary antibiotics.

#### Cell lines

Hybrid Aspen (poplar) CesA8 was expressed in *Spodoptera frugiperda* (Sf9) cells infected with recombinant baculovirus (pACEBac1). SF9 cells were grown in ESF921 medium (Expression systems) at 27°C and cells were harvested 72 h after the infection when cell viability was dropped down to 70%.

### METHOD DETAILS

#### Mutagenesis

The BcsA gating loop (F503A/I, V505A/L, T506A/S, K508A/R) and R382A/F mutants were generated from the wild-type (WT) construct as described earlier^7^ via QuikChange mutagenesis. The WT type construct contains both the *Rhodobacter sphaeroides* cellulose synthase (bcs)A and bcsB genes expressed in pETDuet-1 vector, wherein BcsA was expressed with a C-terminal dodeca-histidine tag. Similarly, the hybrid aspen CesA8 mutants were generated from an existing WT pACEBac-CesA8 plasmid^19^ by the same method. Not all mutations generated for BcsA were also introduced into CesA8. The CesA8 mutants were designed based on activity results obtained for the BcsA mutants.

All the oligonucleotides used in generating the BcsA and CesA8 mutants are provided in Table S5.

#### Expression and purification of CesA8 and its variants in SF9 insect cells

Expression of CesA8 and its mutants was performed in SF9 insect cells and the purification was carried out as described previously,^19^ with some modifications. Briefly, the cell pellet from 1–1.5 L of culture was resuspended in the modified buffer [Buffer A: 20 mM Tris pH 7.5, 100 mM NaCl, 5 mM sodium phosphate, 5 mM sodium citrate, 1 mM TCEP] containing the detergents 1% lauryl maltose neopentyl glycol (LMNG, Anatrace) and 0.2% cholesteryl hemisuccinate (CHS, Anatrace), supplemented with protease inhibitor cocktail (0.8 μM Aprotinin, 5 μM E–64, 10 μM Leupeptin, 15 μM Bestatin-HCl, 100 μM AEBSF-HCl, 2 mM Benzamidine-HCl and 2.9 mM Pepstatin A). The entire mixture was lysed with 20 strokes of a tight fitting 100 mL Dounce homogenizer. After solubilization at 4°C for 1 h, the insoluble material was removed by centrifugation at 42,000 rpm for 45 min in a Ti45 rotor. All subsequent steps including the exchange of detergent from LMNG-CHS to 0.02% glycol-diosgenin (GDN, Anatrace) during the wash buffers were carried out as described before,^19^ except for the omission of size exclusion chromatography. Instead, a dialysis step was performed wherein the Ni-NTA eluent was concentrated to approximately 5 mL using a 100-kDa spin concentrator (Millipore) and then dialyzed overnight against Buffer A containing 0.02% GDN. The following day, the dialyzed protein was concentrated to roughly 1.6–1.9 mg/mL and flash-frozen in small aliquots in liquid nitrogen and then stored at −80°C until further use.

#### Expression and preparation of inverted membrane vesicles of *Rhodobacter sphaeroides* BcsA-B complex and its variants

Expression and inverted membrane vesicle (IMV) preparation was carried out as described previously^6,10^ for the wild-type BcsA-B complex. Briefly, the BcsA-B complex was expressed in *E*. *coli* Rosetta 2 cells in auto-induction medium. The 2 L cell pellet obtained for each protein construct was resuspended in Resuspension Buffer (RB) containing 20 mM Tris pH 7.2, 100 mM NaCl, and 10% Glycerol, supplemented with 1 mM phenylmethylsulfonyl fluoride (PMSF). Cells were lysed in a microfluidizer followed by centrifugation at 12,500 rpm in a Beckman JA-20 rotor for 20 min. The supernatant was carefully recovered and roughly 25 mL was layered over a 1.8 M sucrose cushion made in RB buffer, followed by centrifugation at 42,000 rpm for 120 min in a Ti45 rotor. The dark brown ring formed at the sucrose cushion was carefully withdrawn, diluted 5-fold in the RB buffer, and finally, the membrane vesicles were sedimented via centrifugation at 42,000 rpm for 90 min in a Ti45 rotor. The pellet fraction was rinsed with RB buffer, resuspended in 1 mL RB, and homogenized using a no. 6 paintbrush followed by douncing in a 2 mL grinder. The vesicles were aliquoted in small quantities and flash frozen in liquid N_2_ until further use. All the steps were performed at 4°C.

#### Normalization of protein levels for activity assays

To normalize the concentration of BcsA amongst the WT and mutant IMVs for performing the activity assays, freeze-thawed IMVs were treated with 2% SDS to aid in solubilization and proper migration during SDS-PAGE. Gel loading samples were made from these solubilized IMVs and western blotting against the BcsA-His tag was performed. The band intensities after Western blotting were used to calibrate the amounts of IMVs used for activity assays.

CesA8 protein concentrations for WT and its mutants were normalized based on quantitative SDS-PAGE analysis of the purified proteins. The gel was imaged after Coomassie-staining and further analyzed using LI-COR Odyssey Imaging system to compare the band intensities.

### *In vitro* cellulose synthesis

For *Rhodobacter* BcsA-B, WT and mutant IMVs were used for assaying cellulose biosynthetic activity as described.^10^ In the beginning, a time course was conducted using the wild-type BcsA-B IMVs to find an incubation time in the linear phase of product accumulation. Synthesis reaction was performed by incubating IMVs with 5 mM UDP-Glucose and 0.25 μCi of UDP-[^3^H]-glucose, 30 μM cyclic-di-GMP (c-di-GMP) and 20 mM MgCl_2_ in RB buffer lacking glycerol. Here, cellulose biosynthesis reaction was monitored for different time periods starting from 0 to 180 min. Aliquots were spotted onto Whatman-2MM chromatography paper and developed by descending paper chromatography using 60% ethanol. The polymer retained at the origin was quantified by scintillating counting. Based on this time course, all standard reactions were carried out at 37°C for 30 min. All reactions were performed in triplicate and error bars represent deviations from the means.

For CesA8, WT and mutants, the purified, micelle solubilized protein was used for synthesis reactions. The assays were performed as described previously.^19^ Briefly, 5 μM of purified protein was incubated with 5 mM UDP-Glucose and 0.25 μCi of UDP-[^3^H]-glucose in 20 mM MgCl_2_ in buffer A containing 0.02% GDN. Then, the reactions were spotted onto Whatman-2MM paper, which was developed in an aqueous solution of 60% ethanol. The product retained at the origins was quantified by scintillation counting. As for assays with the bacterial enzyme, we determined a suitable assay time point during the linear product accumulation using the WT and mutants (V853L and T854S). Based on this time course, all subsequent reactions were performed for 30 min at 30°C. To confirm the formation of authentic cellulose, enzymatic degradations of the *in vitro* synthesized glucan were performed using a commercial cellulase (endo-(1,4)-β-glucanase E-CELTR; Megazyme), wherein 5U of the enzyme was added at the beginning of synthesis reaction. All reactions were performed in triplicate and error bars represent deviations from the means.

### Cryo-EM data collection

Ligand bound CesA8 complexes were generated by adding 20 mM MgCl_2_ and 5 mM UDP or UDP-Glc to the purified protein followed by an incubation for 30 min on ice prior to cryo grid preparation. Cryo-EM analyses were performed as described before.^19^ In short, 2.5 μL aliquot was applied to a glow-discharged (in the presence of amylamine) C-flat 400 mesh 1.2/1.3 holey carbon grid (Electron Microscopy Sciences), blotted with Vitrobot Mark IV (FEI, Thermo Fisher Scientific) with force 7 for 12–14 s at 4°C, 100% humidity, and flash frozen in liquid ethane. Grids were screened in-house for optimal ice thickness and particle distribution. High quality datasets were collected at the Brookhaven National Laboratory for BioMolecular Structure (LBMS) on a Titan Krios G3i equipped with an X-FEG electron source, Gatan K3 direct electron detector, and BioQuantum energy filter. Movies were collected in super-resolution mode with a pixel size of 0.4125 and 0.88 Å for the UDP-Glc and UDP-bound complexes, respectively. All movies were collected in counting mode at a magnification of 105,000k and 81K, respectively, and defocus range from −2.3 to −0.8 μm, with a total dose of 51e^−^/Å^2^.

### Data processing

Cryo-EM data processing followed a similar workflow in cryoSPARC^31^ as previously described.^19^ Movies were full-frame motion corrected followed by CTF estimation. Exposures were manually curated based on estimated resolution, defocus, and drift as well as ice contamination.

Initial templates for particle picking were generated using ‘blob picking’ with inner and outer particle diameters of 200 and 350 Å. Particles were extracted with a box size of 600 pixels and Fourier cropped to a box size of 150 pixels. Following 2D classification, selected class averages were used for template-based particle picking. The new particle stack was inspected, extracted with 4-fold Fourier cropping, and classified in 2 and 3-dimensions. The best particles of the UDP-Glc-bound dataset were re-extracted using a 640 pixels box and Fourier cropped to a 320 pixels box. For the UDP complex, final particle stack was extracted at a box size of 400 pixels without cropping. Refinements followed standard non-uniform refinement with C3 symmetry.

#### Model building

PDB entry 6WLB was used as an initial model. The previous CesA8 trimer structure was rigid body docked into the EM map in Chimera^35^ and manually adjusted in Coot.^32^ UDP and UDP-alpha-D-Glc were placed and manually refined in Coot. The model was refined in phenix:refine^33^ without imposing non-crystallographic symmtery (NCS). Coordinates and EM maps have been deposited at the Protein DataBank under accession codes 8G27 and 8G2J.

#### Molecular dynamics system construction

The MD workflow started from the cryo-EM structure of poplar CesA8 with a bound UDP, coordinated Mg^2+^, and cellopentaose. MD simulations were performed with a monomeric CesA8 construct. Rather than a TM7 (932–958) of the same CesA8 monomer, this monomeric construct contained the TM7 of the adjacent CesA8 subunit to complete its TM channel architecture. Then, the unresolved gating loop was modeled by analogy to BcsA (PDB: 5EIY), representing an inserted state of the gating loop. Subsequently, the SWISS-MODEL web server was used to generate initial coordinates for the remaining residues of the gating loop (847–848 and 856–865); SWISS-MODEL employs the ProMod3 homology modeling engine, which uses a database of structural fragments derived from the Protein Data Bank or falls back to a Monte Carlo approach to generate coordinates.^43–45^ A UDP-Glc-bound case was also prepared for MD simulation, based on the experimentally determined UDP-alpha-D-Glc-bound CesA8 structure described herein. The AMBER 2019 software package^36^ was used for all subsequent system construction, force field implementation, and simulation tasks. The simulation environment was assembled around each initial model (CesA8 monomer with UDP/UDP-Glc, Mg^2+^, and cellopentaose). Specifically, AMBER’s packmol-memgen, with Packmol 18.169, was used to add a homogeneous dioleoylphosphatidylcholine (DOPC) phospholipid bilayer, water, and 0.15 M NaCl; unless indicated, default settings such as the padding distances for the lipid bilayer and water around the solute were used.^40,46^ For each of the two cases (UDP- or UDP-Glc-bound), three independent systems were prepared by using a pseudo-random seed during the packmol-memgen system assembly stage, leading to a total of six modeled systems.

#### MD force fields

AMBER’s tleap was then used to apply selected force fields (set of potential energy expressions, parameters, and structural libraries) to the constructed systems. The following force fields were used: ff14SB (CesA protein),^47^ Lipid17 (DOPC),^36,48^ GLYCAM06j (cellopentaose),^49^ TIP3P model (water),^50^ and the 12–6-4 Lennard-Jones (LJ) set of the Li-Merz monovalent and divalent ion parameters for TIP3P (Na^+^, Cl^−^, Mg^2+^).^51–53^ Base parameters for the uridine and phosphate groups of UDP were provided through AMBER’s ‘‘parm10.dat’’, which includes the OL3 parameters for RNA.^54^ Parameters for the glucosyl group of UDP-Glc were provided through GLYCAM06. The parameters describing the connection between the beta phosphate and the glucosyl group were assigned by analogy to GLYCAM06; specifically, one bond parameter, four angle parameters, and seven dihedral parameters were added based on methyl sulfate, ethyl sulfate, methoxy alkanes, and ether alcohols (Table S4). UDP parameters based on GLYCAM93 have been developed previously^55^; while this previous work was used as a general reference point, we sought to remain consistent with the newer GLYCAM06 and thus adopted comparable parameters directly from GLYCAM06. Furthermore, a modification was applied to the 12–6 LJ parameters of the hydroxyl hydrogen atom type ‘‘HO’’ as used in AMBER’s ‘‘all_modrna08.frcmod’’^56^ (Table S4); this was deemed necessary to avoid a known potential issue when using hydroxyl hydrogen atom types with Rmin and epsilon parameters of zero.

#### MD partial charges

For both UDP and UDP-Glc, the net charge was considered as −2.0 e, that is, a single protonation on the beta phosphate of UDP and no protonation of the UDP-Glc phosphates. This selected protonation state of UDP is based on log(K_a_) values that have been reported for adenosine diphosphate, ADP (7.02, 4.19, and 0.9 for single, double, and triple protonation of the phosphates) via ^1^H NMR chemical shift data and non-linear regression.^57^ The selected protonation state of UDP-Glc is based on pK_a_ values that have been reported for UDP-GlcNAc – an N-acetyl derivative of UDP-Glc (6.6 and 6.3 for the alpha and beta phosphates, respectively) via ^31^P NMR chemical shift data^58^; thus, above these pKa values, one might expect a larger population of the phosphate-deprotonated UDP-Glc species. Partial charges for UDP and UDP-Glc (UDP moiety only) were obtained from project F-90 of the RESP ESP Charge Database (R.E.DD.B.)^59^; fragments 1 (‘‘POP’’, alpha and beta phosphates for UDP-Glc), 2 (‘‘P1’’, alpha phosphate for UDP), 3 (‘‘P1M’’, beta phosphate for UDP), and 47 (‘‘U5’’, uridine) of this project were used. Partial charges for the glucosyl group of UDP-Glc were obtained from a terminal alpha-D-glucose unit of GLYCAM06 (entry ‘‘0GA’’ in its library). However, for the carbon at the reducing end of this glucosyl group linked to the beta phosphate, a charge adjustment of +0.0102 was applied to this glucosyl ‘‘C1’’ atom to provide an integer net charge of −2.0 for the UDP-Glc molecule; within the modular or fragment-based framework of GLYCAM06 and related force fields, such charge adjustments at linking atoms have been used for carbohydrate derivatives, e.g., O-acetyl, O-methyl, O-sulfate, and N-sulfate modifications, and are applicable to other derivatives.

#### MD simulations

The MD simulation protocol employed is based on those used previously to simulate protein and lipid systems.^28,60–62^ Specifically, a 10-stage protocol was used including up to 10,000 steps of energy minimization (5,000 steepest descent steps followed by conjugate gradient steps), gradual NVT heating to 300 K over 100 ps, NVT equilibration at 300 K for 200 ps, NPT equilibration at 300 K and 1 atm for 800 ps, and 500 ns of production MD at 300 K and 1 atm.^60^ A cutoff of 1.0 nm, periodic boundary conditions (PBCs) in all directions, particle-mesh Ewald (PME),^63^ and long-range Lennard-Jones correction^36^ were used for all stages. The timestep was initially 1.0 fs but was increased to 2.0 fs during the last 700 ps of the NPT equilibration stages and during the NPT production stage. The SHAKE algorithm^64^ was used to constrain bonds involving hydrogen atoms during all stages after the energy minimization stage. Step-wise-reduced harmonic positional restraints were used for all protein atoms (10, 5.0, 2.5, 1.0, 0.5, 0.1 kcal/mol/Å^2^) and for the lipid head group phosphorus atoms (2.5, 2.5, 1.0, 0.5, 0.1, 0.0 kcal/mol/Å^2^)^62^ up to the end of the first 400 ps of the NPT equilibration, after which there were no restraints. The Langevin thermostat^65^ (collision frequency = 1.0 ps^−1^) and Berendsen barostat^66^ (coupling constant = 1.0 ps, compressibility = 44.6*10^−6^ bar^−1^, anisotropic scaling) were used, where applicable. Above, ‘‘NVT’’ and ‘‘NPT’’ refer to thermodynamic ensembles described by a constant number of particles-fixed volume-regulated temperature and constant number of particles-regulated pressure-regulated temperature, respectively. AMBER’s CPU-only PMEMD and single precision-fixed point/SPFP GPU-accelerated PMEMD programs were used for the energy minimization and subsequent simulation stages, respectively, where CPU = central processing unit and GPU = graphics processing unit.^67,68^ These simulations were carried out with Exxact Corporation servers equipped with NVIDIA GeForce GTX GPUs. For each of the two cases (UDP- or UDP-Glc-bound), three independent MD simulations were conducted; further independence from the system assembly stage was afforded by also using a pseudo-random seed for the Langevin thermostat during the simulations.

#### MD analysis

Contacts between residues of the CesA8 gating loop (847–869) and the ligand groups (uridine, alpha phosphate, beta phosphate, Mg^2+^, and glucosyl group, as applicable) were analyzed using PyContact 1.0.4^37^ with MDAnalysis 0.20.1.^38,39^ Contact criteria included considering heavy atoms only and a contact distance cutoff of 0.5 nm.^37^ Contact metrics were calculated and accumulated over atom-atom contacts to obtain residue-residue contact data, where ‘‘residue’’ here can refer to an amino acid residue or a ligand group as listed above. Contact metrics of interest included the mean contact score (based on a distance-weighted sigmoidal function), mean contact lifetime (ns), and total contact time (ns and %). The contact analysis was conducted using 1,000 evenly sampled frames from each simulation coordinate trajectory representing 500 ns of time. The contact metrics were then averaged over the three independent simulations for each case.

### QUANTIFICATION AND STATISTICAL ANALYSIS

Average and standard deviation values were determined using AVERAGE and STDEV functions in Excel, and data plotting was performed using Microsoft Excel and GraphPad Prism 6.0.

## Supplementary Material

MMC1

## Figures and Tables

**Table:** KEY RESOURCES TABLE

REAGENT or RESOURCE	SOURCE	IDENTIFIER
Antibodies		

Penta ∙ His Antibody, BSA-free	Qiagen	Cat# 34660; RRID: AB_2619735
Anti-Mouse IgG (H&L) (Goat) Antibody DyLight 800 Conjugated	Rockland	Cat# 610-145-002; RRID:AB_10703265

Bacterial and virus strains		

Rosetta 2(DE3) Singles Competent Cells	Novagen	Cat# 71400
NEB^®^ 5-alpha Competent *E. coli* (High Efficiency)	New England Biolabs	Cat# C2987

Chemicals, peptides, and recombinant proteins		

Uridine 5ʹ-diphosphate disodium salt hydrate	Sigma-Aldrich	Cat# 94330; CAS: 27821-45-0
Uridine 5ʹ-diphosphoglucose disodium salt hydrate from *Saccharomyces cerevisiae*	Sigma-Aldrich	Cat# U4625; CAS: 28053-08-9
Uridine diphospho-D-glucose, [6-^3^H]-, 250 μCi	PerkinElmer	Cat# NET1163250UC
Lauryl Maltose Neopentyl Glycol	Anatrace	Cat# NG310
Cholesteryl Hemisuccinate Tris Salt	Anatrace	Cat# CH210
Glyco-diosgenin (GDN)	Anatrace	Cat# GDN101
Aprotinin, Bovine Lung	AG Scientific	Cat# A-1420; CAS: 9087-70-1
Leupeptin hemisulfate	Adooq	Cat# A14145; CAS: 103476-89-7
Bestatin hydrochloride	APExBIO	Cat# A8621; CAS: 65391-42-6
AEBSF HCl	AG Scientific	Cat# A-1018; CAS: 30827-99-7
Pepstatin A	Adooq	Cat# A14262; CAS: 26305-03-3
Benzamidine Hydrochloride Monohydrate	GoldBio	Cat# B-050; CAS: 1670-14-0
E-64	APExBIO	Cat# A2576; CAS: 66701-25-5
Phenylmethanesulphonyl fluoride	VWR	Cat# 97064; CAS: 329-98-6
FuGENE HD Transfection Reagent	Promega	Cat# E2311
ESF 921 Insect Cell Culture Medium, Protein Free	Expression Systems	Cat# 96-001-01
Fetal Bovine Serum, Heat Inactivated	Sigma Aldrich	Cat# 12306C

Critical commercial assays		

Cellulase (*endo*-1,4-β-D-glucanase) (*Trichoderma longibrachiatum*)	Megazyme/Neogen	Cat# E-CELTR

Deposited data		

Structure of homotrimeric poplar cellulose synthase isoform 8	Purushotham et al.^19^	PDB: 6WLB
Coordinates of UDP bound CesA8	This study	PDB: 8G27
Cryo-EM structure of UDP bound CesA8	This study	EMD: 29678
Coordinates of UDP-Glucose bound CesA8	This study	PDB: 8G2J
Cryo-EM structure of UDP-Glucose bound CesA8	This study	EMD: 29679

Experimental models: Cell lines		

*Spodoptera frugiperda* Sf9	ATCC	Cat# CRL-1711; RRID: CVCL_0549

Oligonucleotides		

Shown in Table S5	This study	N/A

Recombinant DNA		

pETDuetBcsA_WT-His_12_-BcsB	Morgan et al.^7^	N/A
pETDuetBcsA_F503A-His_12_-BcsB	This study	N/A
pETDuetBcsA_F503I-His_12_-BcsB	This study	N/A
pETDuetBcsA_V505A-His_12_-BcsB	This study	N/A
pETDuetBcsA_V505L-His_12_-BcsB	This study	N/A
pETDuetBcsA_T506A-His_12_-BcsB	This study	N/A
pETDuetBcsA_T506S-His_12_-BcsB	This study	N/A
pETDuetBcsA_K508A-His_12_-BcsB	This study	N/A
pETDuetBcsA_K508R-His_12_-BcsB	This study	N/A
pETDuetBcsA_R382A-His_12_-BcsB	This study	N/A
pETDuetBcsA_R382F-His_12_-BcsB	This study	N/A
pACEBac1-His_12_-*Ptt*CesA8_WT	Purushotham et al.^19^	N/A
pACEBac1-His_12_-CesA8_R717A	This study	N/A
pACEBac-His_12_-CesA8_F851I	This study	N/A
pACEBac-His_12_-CesA8_V853L	This study	N/A
pACEBac-His_12_-CesA8_T854S	This study	N/A
pACEBac-His_12_-CesA8_T854A	This study	N/A
pACEBac-His_12_-CesA8_K856R	This study	N/A

Software and algorithms		

Cryosparc v.2.8	Punjani et al.^31^	https://cryosparc.com/
Coot v0.9.6	Emsley and Cowtan^32^	https://www2.mrc-lmb.cam.ac.uk/personal/pemsley/coot/
Phenix	Adams et al.^33^	http://www.phenix-online.org/
PyMol v2.5.4	PyMol^34^	https://pymol.org/2/
Chimera 1.15	Pettersen et al.^35^	https://www.cgl.ucsf.edu/chimera/
Origin 2021b	OriginLab^30^	https://www.originlab.com/
GraphPad Prism 6.0	GraphPad	https://www.graphpad.com/scientific-software/prism/
Amber 2019	Case et al.^36^	https://ambermd.org/
PyContact 1.0.4	Scheurer et al.^37^	https://pycontact.github.io/
MDAnalysis 0.20.1	Michaud-Agrawal et al.^38^; Gowers et al. ^39^	https://www.mdanalysis.org/
Packmol 18.169	Martínez et al.^40^; Schott-Verdugo and Gohlke^46^	https://m3g.github.io/packmol/
Clustal Omega	Sievers et al.^41^	https://www.ebi.ac.uk/Tools/msa/clustalo/

Other		

Superose 6 Increase 10/300 GL (Bed dimensions 10 X 300)	Cytiva	Cat# 29091596
HisPur Ni-NTA Resin	ThermoFisher Scientific	Cat# 88222

## Highlights

- Cryo-EM structure of UDP-glucose-bound poplar cellulose synthase-8
- Site-directed mutagenesis analyzes the function of a flexible gating loop
- Conserved gating loop residues are necessary for cellulose biosynthesis
- Molecular dynamics simulations support persistent gating loop-substrate interactions
