# Supplementary material for: Insights into substrate coordination and glycosyl transfer of poplar cellulose synthase-8
Source: Structure. Author manuscript; Available in PMC 2023 Oct 23. (PMC10592267; doi:10.1016/j.str.2023.07.010)
Supplement: MMC1 [file NIHMS1921124-supplement-MMC1.pdf]

Structure, Volume *31*

Supplemental Information

Insights into substrate coordination and glycosyl transfer of poplar cellulose synthase-8

Preeti Verma, Albert L. Kwansa, Ruoya Ho, Yaroslava G. Yingling, and Jochen Zimmer

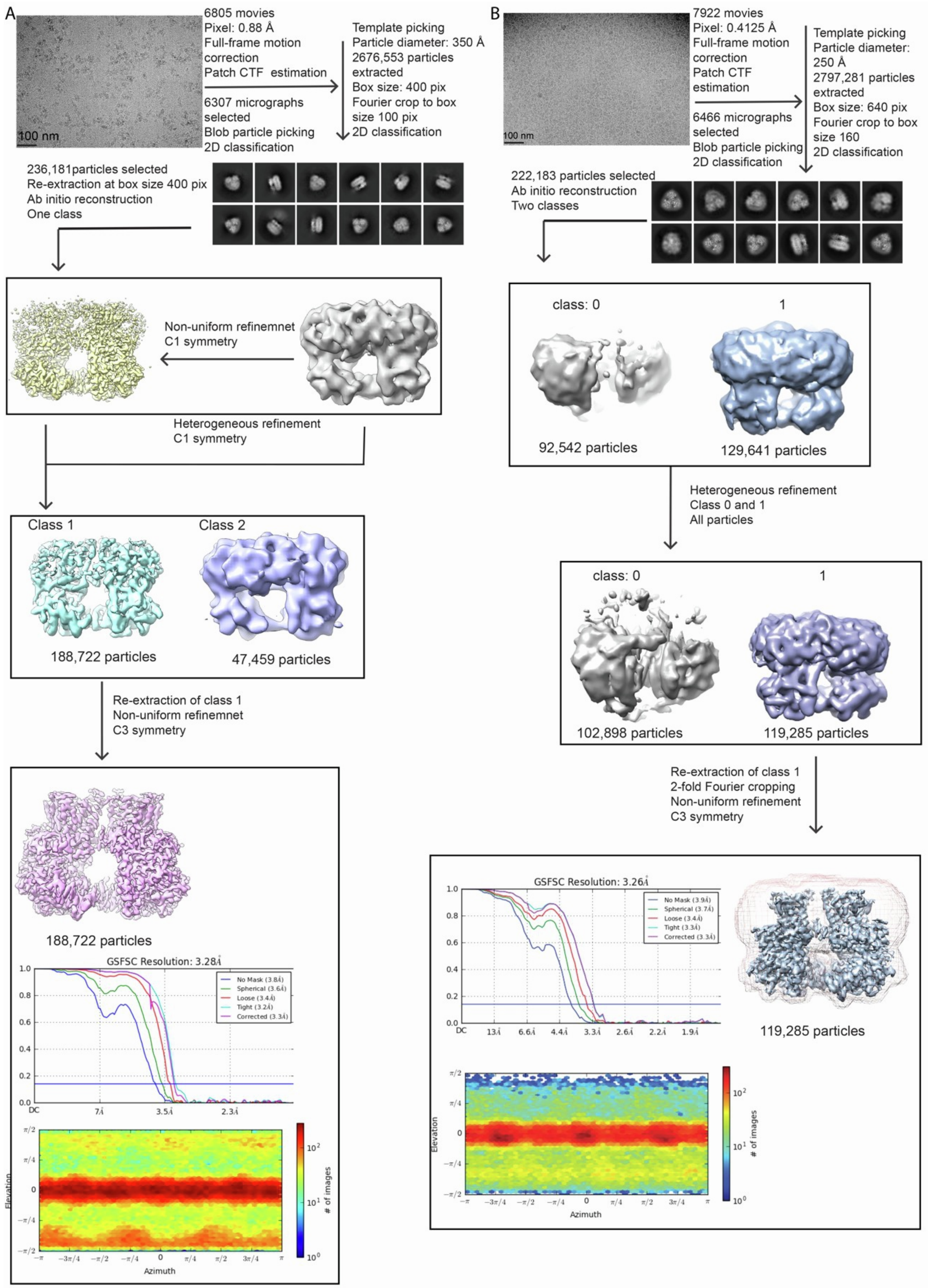
A
6805 movies
Pixel: 0.88 Å
Full-frame motion correction
Patch CTF estimation
6307 micrographs selected
Blob particle picking
2D classification
100 nm
Template picking
Particle diameter: 350 Å
2676,553 particles extracted
Box size: 400 pix
Fourier crop to box size 100 pix
2D classification
236,181particles selected
Re-extraction at box size 400 pix
Ab initio reconstruction
One class
Non-uniform refinemnet
C1 symmetry
Heterogeneous refinement
C1 symmetry
Class 1
Class 2
188,722 particles
47,459 particles
Re-extraction of class 1
Non-uniform refinemnet
C3 symmetry
188,722 particles
GSFSC Resolution: 3.28Å
No Mask (3.8Å)
Spherical (3.6Å)
Loose (3.4Å)
Tight (3.2Å)
Corrected (3.3Å)
Elevation
Azimuth
# of images
B
7922 movies
Pixel: 0.4125 Å
Full-frame motion correction
Patch CTF estimation
6466 micrographs selected
Blob particle picking
2D classification
100 nm
Template picking
Particle diameter: 250 Å
2797,281 particles extracted
Box size: 640 pix
Fourier crop to box size 160
2D classification
222,183 particles selected
Ab initio reconstruction
Two classes
class: 0
1
92,542 particles
129,641 particles
Heterogeneous refinement
Class 0 and 1
All particles
class: 0
1
102,898 particles
119,285 particles
Re-extraction of class 1
2-fold Fourier cropping
Non-uniform refinement
C3 symmetry
GSFSC Resolution: 3.26Å
No Mask (3.9Å)
Spherical (3.7Å)
Loose (3.4Å)
Tight (3.3Å)
Corrected (3.3Å)
119,285 particles
Elevation
Azimuth
# of images

Figure S1. Cryo-EM data processing workflow, related to Figure 1. (**A**) UDP bound CesA8. (**B**) UDP-Glc bound CesA8.

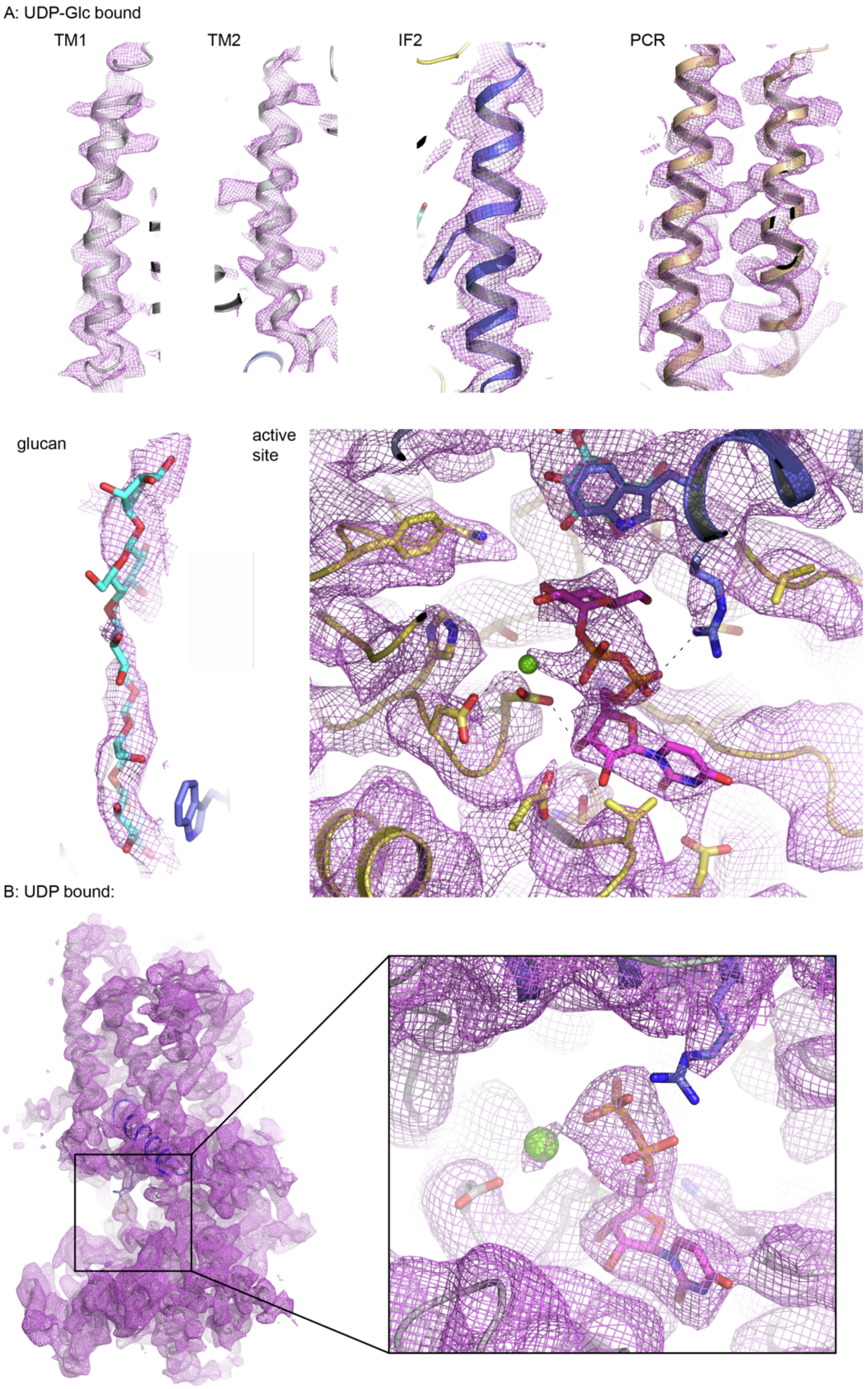

Figure S2. Examples of cryo-EM map qualities, related to Figure 1. (**A**) UDP-Glc bound CesA8 and (**B**) UDP bound CesA8.

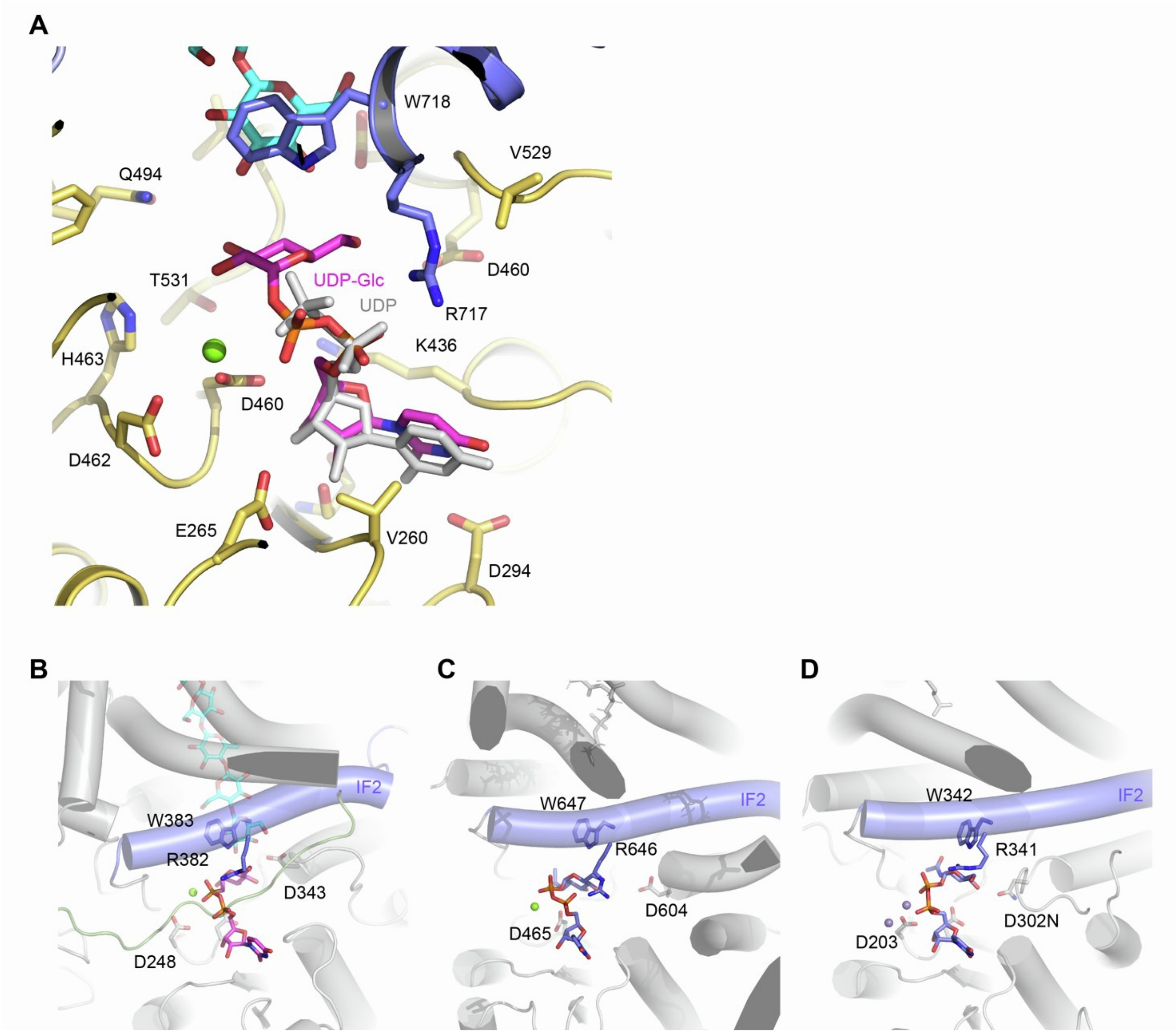

Figure S3. Comparison of substrate binding poses, **related to Figure 1. (A)** Overlay of UDP and UDP-Glc substrate poses bound to poplar CesA8. **(B-D) Substrate binding to** *Rhodobacter sphaeroides* BcsA (B, PDB: 5EIY), chitin synthase (C, PDB: 7STM), and hyaluronan synthase (D, PDB: 7SP8).

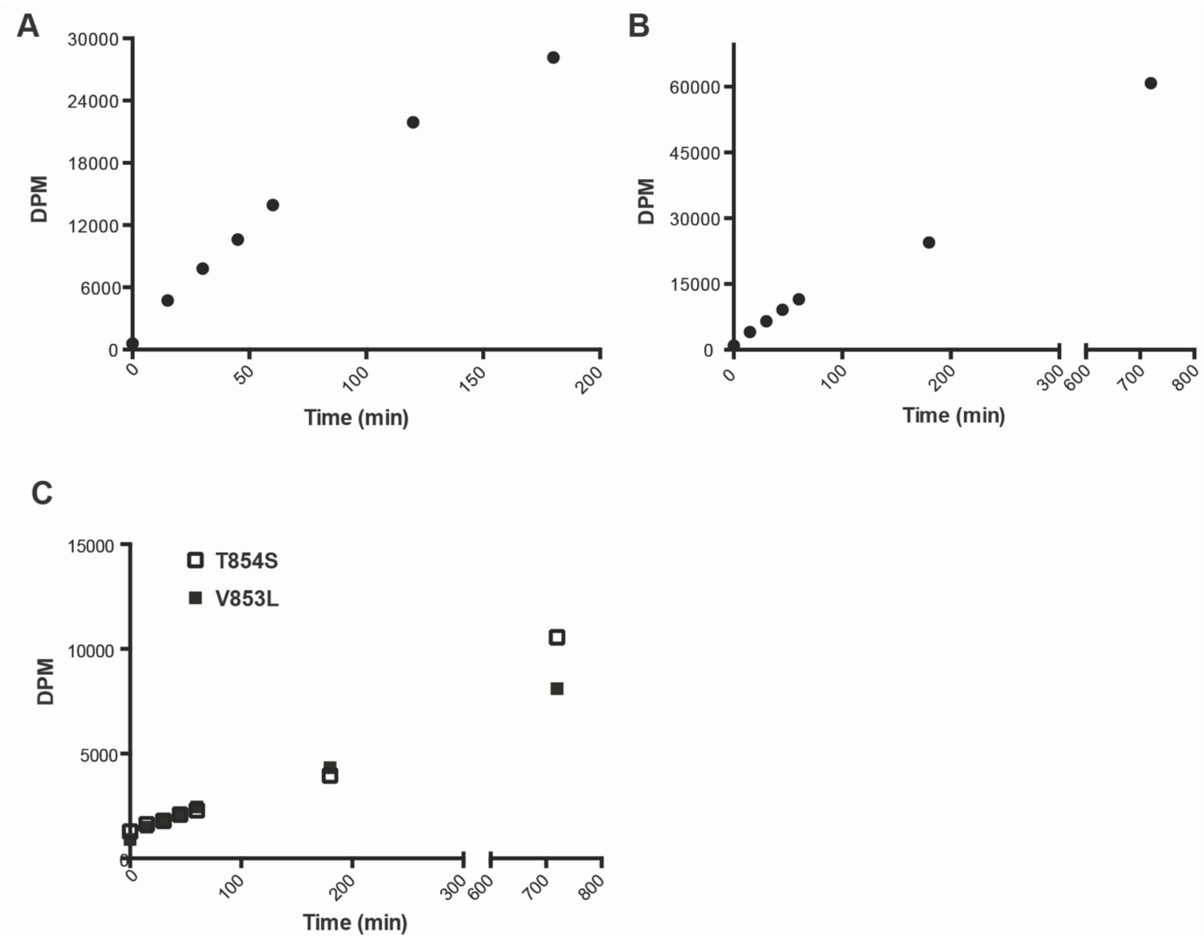

Figure S4. Time course of cellulose biosynthesis, related to Figure 2. (A) Cellulose synthesis reaction for wild-type (WT) BcsA-B IMVs was performed at 37ºC for different time periods starting from 0 to 180 min. At each time interval, 20 µl of reaction mixture was withdrawn, and 2% SDS was added to terminate the synthesis reaction. The products were quantified by scintillation counting. **(B)** Time course of product accumulation for wildtype (WT) poplar CesA8. CesA8 synthesis reactions were incubated at 30ºC and at each indicated time interval, a sample was withdrawn and spotted onto Whatman-2MM blotting paper for quantification. DPM: Disintegrations per minute. **(C)** As for panel B but with the indicated CesA8 mutants.

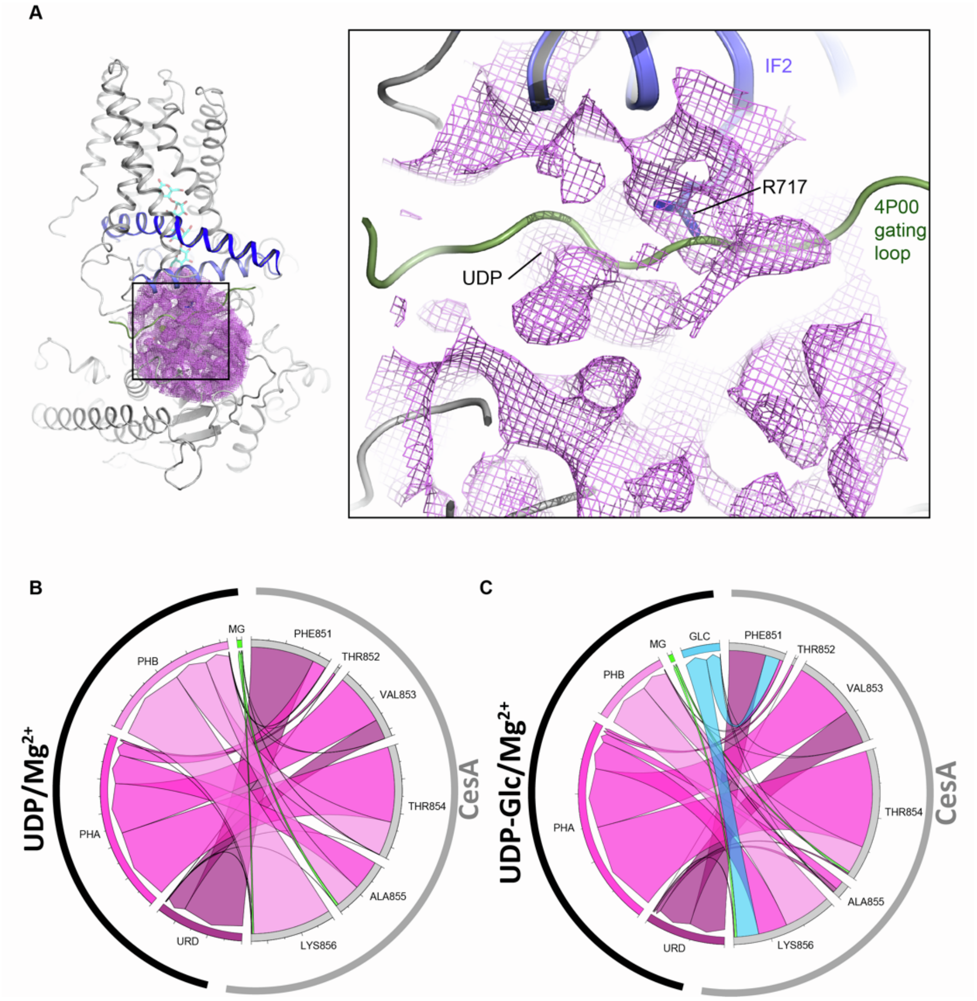

Figure S5. CesA8's gating loop interacts with the nucleotide at the active site, related to Figure 3. (**A**) Shown is the cryo-EM map of the UDP-Glc-bound CesA8 complex at a low contour level. The green ribbon indicates the gating loop position in UDP-bound *Rhodobacter* BcsA (PDB: 4P00). (**B** and **C**) Interactions of CesA8's gating loop with UDP or UDP-Glc and Mg^{2+}. Chord diagrams based on mean contact scores between gating loop residues of CesA8, groups of UDP/UDP-Glc (URD = uridine, PHA = alpha phosphate, PHB = beta phosphate, and GLC = alpha-D-glucosyl group), and the magnesium ion (MG). Mean contact score refers to a mean over 1,000 frames corresponding to 500 ns of simulated time, and the mean scores were averaged from three independent simulations. The widths of the nodes (arcs) and links (arrows) are weighted by the mean contact scores, and the links are colored based on their UDP/UDP-Glc/Mg^{2+} destination arcs. (Generated with Origin [S1]).

Table S1. EM and model stats, related to Figure 1.

Cryo-electron microscopy data collection and processing		
	**UDP-Glc bound**	**UDP bound**
Microscope	FEI Titan Krios G3i	FEI Titan Krios G3i
Voltage (keV)	300	300
Camera	Gatan K3	Gatan K3
Energy Filter	BioQuantum	BioQuantum
Pixel size (Å)	0.412	0.88
Defocus range (µm)	-2.3 – -0.8	-2.3 – -0.8
Magnification (nominal)	105,000	81,000
Electron exposure (e^-/pix/s)	15	15
Exposure rate (e^-/Å^2)	51	50
Frames per movie	50	40
Energy filter slit width (eV)	20	10
Automation software	EPU	EPU
Micrographs used	6466	6307
Extracted particles	2,797,281	2,676,553
Particles in final 3D refinement	119,285	188,722
Resolution No mask (Å)	3.9	3.8
Resolution Spherical	3.7	3.6
Resolution Loose	3.4	3.4
Resolution Tight	3.3	3.3
Resolution Corrected	3.3	3.3
Sharpening B-factor (Å2)	-130	-129
EMDB ID		
**Coordinate Refinement and Validation**		
Refinement program	Phenix (Real-space refinement)	Phenix (Real-space refinement)
Number of protein atoms (non-H)	34467	34562
Number of ligands	CE5: UDP-Glc:MG3:3:3	CE5:UDP:Mg 3:3:3
RMSD bond (Å)	0.002	0.002
RMSD angle (º)	0.595	0.562
Ramachandran favored (%)	95.14	94.62
Ramachandran allowed (%)	4.68	5.19
Ramachandran outlier (%)	0.19	0.19
All-atom clash score	8.67	5.06
MolProbity Score	1.8	1.64
B-factors (min/max/mean)		
Protein	34/153/82	8/211/92
Overall correlation coefficient		
CC (mask)	0.75	0.83
CC (box)	0.59	0.68
CC (peaks)	0.49	0.6
CC (volume)	0.73	0.81
Mean CC for ligands	0.6	0.67
PDB ID	8G2J	8G27

Table S2. Contact pairs between CesA protein residues and ligand groups (UDP and Mg^{2+}), and their contact metrics, related to Figure 3 and S5. The ligand groups include URD (uridine), PHA (alpha phosphate), PHB (beta phosphate), and MG (Mg^{2+}). Mean score and mean lifetime represent means over 1,000 frames corresponding to 500 ns of simulated time, and all metrics were averaged from three independent simulations. The contacts are sorted by CesA residue ID number and then by mean score. The numerical columns, mean score to total time (%), are colored with a green-yellow-red scale from highest to lowest.

Residue	Ligand group	Mean score	Mean lifetime (ns)	Total time (ns)	Total time (%)
PHE851	URD	3.86	244.98	494.00	98.80
PHE851	PHA	0.83	25.75	404.67	80.93
PHE851	PHB	0.04	1.68	59.00	11.80
THR852	PHA	0.13	8.33	419.17	83.83
THR852	URD	0.01	1.49	25.00	5.00
THR852	PHB	0.00	0.58	3.50	0.70
VAL853	PHA	3.19	204.53	489.33	97.87
VAL853	URD	1.21	21.25	458.50	91.70
VAL853	PHB	0.01	1.23	57.67	11.53
VAL853	MG	0.00	0.66	29.50	5.90
THR854	PHA	4.59	226.10	476.50	95.30
THR854	PHB	2.08	184.33	466.00	93.20
THR854	MG	0.02	1.87	159.75	31.95
THR854	URD	0.00	0.58	18.00	3.60
ALA855	PHB	1.52	13.05	244.83	48.97
ALA855	PHA	1.84	199.78	360.33	72.07
ALA855	URD	0.01	1.06	7.17	1.43
ALA855	MG	0.15	18.51	188.17	37.63
LYS856	PHB	4.78	14.03	226.00	45.20
LYS856	MG	0.10	4.61	164.67	32.93
LYS856	URD	0.05	12.27	159.50	31.90
LYS856	PHA	0.01	0.68	65.50	13.10
ALA857	PHB	0.00	0.66	59.00	11.80
ALA858	URD	0.00	0.25	0.50	0.10
ASP860	URD	0.00	0.50	1.33	0.27
ASP860	PHA	0.00	0.33	1.00	0.20
GLU862	URD	0.00	0.33	1.17	0.23

Table S3. Contact pairs between CesA protein residues and ligand groups (UDP-Glc and Mg^{2+}), and their contact metrics, related to Figure 3 and S5. The ligand groups include URD (uridine), PHA (alpha phosphate), PHB (beta phosphate), GLC (alpha-D-glucosyl group), and MG (Mg^{2+}). Mean score and mean lifetime represent means over 1,000 frames corresponding to 500 ns of simulated time, and all metrics were averaged from three independent simulations. The contacts are sorted by CesA residue ID number and then by mean score. The numerical columns, mean score to total time (%), are colored with a green-yellow-red scale from highest to lowest.

Residue	Ligand group	Mean score	Mean lifetime (ns)	Total time (ns)	Total time (%)
PHE851	URD	1.11	12.20	211.17	42.23
PHE851	GLC	0.44	3.22	109.50	21.90
PHE851	PHA	0.10	2.38	88.83	17.77
PHE851	PHB	0.00	0.80	30.50	6.10
THR852	PHA	0.07	4.14	184.83	36.97
THR852	PHB	0.00	0.33	1.00	0.20
THR852	URD	0.00	0.33	1.00	0.20
VAL853	PHA	1.87	167.98	265.17	53.03
VAL853	URD	1.03	11.05	300.67	60.13
VAL853	GLC	0.00	0.88	8.00	1.60
VAL853	PHB	0.00	0.71	4.17	0.83
THR854	PHA	2.93	169.23	322.33	64.47
THR854	PHB	0.83	57.33	317.50	63.50
THR854	URD	0.04	0.75	34.00	6.80
THR854	GLC	0.00	1.03	17.00	3.40
THR854	MG	0.06	3.21	96.00	19.20
ALA855	PHA	0.27	2.34	125.50	25.10
ALA855	URD	0.12	1.82	55.83	11.17
ALA855	PHB	0.02	1.43	32.17	6.43
ALA855	MG	0.00	0.80	11.00	2.20
ALA855	GLC	0.00	0.33	1.00	0.20
LYS856	PHB	1.45	5.39	206.50	41.30
LYS856	PHA	0.88	6.17	143.67	28.73
LYS856	GLC	0.66	3.55	181.00	36.20
LYS856	MG	0.07	4.51	140.50	28.10
LYS856	URD	0.03	1.07	34.00	6.80
ALA857	URD	0.01	0.79	19.00	3.80
ALA858	URD	0.00	2.00	2.00	0.40
ASP859	URD	0.19	1.92	46.00	9.20
ASP860	URD	0.00	0.25	0.50	0.10

Table S4. Additional force field parameters employed for UDP-alpha-D-Glc assigned by analogy to GLYCAM06, and modified hydroxyl hydrogen "HO" parameters applied broadly based on that described previously for modified nucleic acids, related to Figure 3 and S5. The bond parameters include the bond force constant (kcal/mol/Å^2) and equilibrium bond length (Å). The angle parameters include the angle force constant (kcal/mol/rad^2) and equilibrium angle (degrees). The dihedral parameters include the energy barrier division factor, half of the energy barrier height (kcal/mol), phase angle (degrees), and the dihedral multiplicity; a negative dihedral multiplicity only indicates that there are additional subsequent terms. The Lennard-Jones (LJ) parameters include half of the interatomic separation distance at the LJ energy minimum, Rmin (Å), and the energy-well depth at the energy minimum, ε (kcal/mol).

Name	Atom types & parameters					Source
Bond 1	Cg-OS	285.00	1.46			GLYCAM_06j.dat
Angle 1	H2-Cg-OS	60.00	110.00			GLYCAM_06j.dat
Angle 2	Os-Cg-OS	100.00	112.00			GLYCAM_06j.dat
Angle 3	OS-Cg-Cg	70.00	108.50			GLYCAM_06j.dat
Angle 4	P -OS-Cg	50.0	118.88			GLYCAM_06j.dat
Dihedral 1	OS-Cg-Os-Cg	1	0.96	0.0	-3.	GLYCAM_06j.dat
		1	1.38	0.0	-2.	
		1	1.08	0.0	1.	
Dihedral 2	OS-Cg-Cg-Cg	1	-0.27	0.0	1.	GLYCAM_06j.dat
Dihedral 3	H1-Cg-Cg-OS	1	0.05	0.0	3.	GLYCAM_06j.dat
Dihedral 4	Oh-Cg-Cg-OS	1	-1.10	0.0	-1.	GLYCAM_06j.dat
		1	0.25	0.0	2.	
Dihedral 5	H2-Cg-OS-P	1	0.17	0.0	3.	GLYCAM_06j.dat
Dihedral 6	Os-Cg-OS-P	1	-1.20	0.0	1.	GLYCAM_06j.dat
Dihedral 7	Cg-Cg-OS-P	1	-1.20	0.0	1.	GLYCAM_06j.dat
LJ 1	HO	0.6000	0.0157			all_modrna08.frcmod

Table S5. Oligonucleotides used for generating BcsA and CesA8 mutants, related to Star Methods. Mutated sequences are indicated with lowercase letters.

Name	Oligonucleotides
BcsA-R382F-Fw	CCTTCATCCAGCAGCGCGGCttcTGGGCCACCGGCATGATGCAG
BcsA-R382F-Rv	CTGCATCATGCCGGTGGCCCAgaaGCCGCGCTGCTGGATGAAGG
BcsA-R382A-Fw	TGGGCGACGGGTATGATGCAGATGCTGCTGCTGAAG
BcsA-R382A-Rv	CGTCGCCCAcgcGCCGCGCTGCTGGATGAAG
BcsA-F503I-Fw	GCCGTTACTGCCAAGGACGAGACGCTGAGCGAG
BcsA-F503I-Rv	AGTAACGGCaatGCGGGCACTGCGCGGCCGCAG
BcsA-F503A-Fw	TGCGGCCGCGCAGTGCCCGCgccGCGGTGACCGCGAAGGACGAGAC
BcsA-F503A-Rv	GTCTCGTCCTTCGCGGTCACCGCggcGCGGGCACTGCGCGGCCGCA
BcsA-V505L-Fw	CCGCGCAGTGCCCGCTTCGCGctgACCGCGAAGGACGAGACGCTG
BcsA-V505L-Rv	CAGCGTCTCGTCCTTCGCGGTcagCGCGAAGCGGGCACTGCGCGG
BcsA-V505A-Fw	GCGCAGTGCCCGCTTCGCGgcgACCGCGAAGGACGAGACGCTG
BcsA-V505A-Rv	CAGCGTCTCGTCCTTCGCGGTcgcCGCGAAGCGGGCACTGCGC
BcsA-T506S-Fw	CGCAGTGCCCGCTTCGCGGTGtccGCGAAGGACGAGACGCTGAG
BcsA-T506S-Rv	CTCAGCGTCTCGTCCTTCGCggaCACCGCGAAGCGGGCACTGCG
BcsA-T506A-Fw	CGCAGTGCCCGCTTCGCGGTGgcgGCGAAGGACGAGACGCTGAGCG
BcsA-T506A-Rv	CGCTCAGCGTCTCGTCCTTCGCcgcCACCGCGAAGCGGGCACTGCG
BcsA-K508R-Fw	GCTTCGCGGTGACCGCGcgcGACGAGACGCTGAGCGAGAAC
BcsA-K508R-Rv	GTTCTCGCTCAGCGTCTCGTCgcgCGCGGTCACCGCGAAGC
BcsA-K508A-Fw	GATGAAACCTTAAGCGAGAACTACATTTCG
BcsA-K508A-Rv	GGTTTCATCggcCGCGGTCACCGCGAAGCGGGC
CesA8-R717A-Fw	CACCAGGTTCTCgcaTGGGCTCTTGGA
CesA8-R717A-Rv	TCCAAGAGCCCAtgcGAGAACCTGGTG
CesA8-F851I-Fw	ATTGATACGAACattACTGTCACAGCA
CesA8-F851I-Rv	TGCTGTGACAGTaatGTTCGTATCAAT
CesA8-V853L-Fw	ACGAACTTTACTctcACAGCAAAAGCA
CesA8-V853L-Rv	TGCTTTTGCTGTgagAGTAAAGTTCGT
CesA8-T854S-Fw	AACTTTACTGTCtcaGCAAAAGCAGCC
CesA8-T854S-Rv	GGCTGCTTTTGCtgaGACAGTAAAGTT
CesA8-T854A-Fw	AACTTTACTGTCgcaGCAAAAGCAGCC
CesA8-T854A-Rv	GGCTGCTTTTGCtgcGACAGTAAAGTT
CesA8-K856R-Fw	ACTGTCACAGCAagaGCAGCCGATGAT
CesA8-K856R-Rv	ATCATCGGCTGCtctTGCTGTGACAGT

Supplemental references

S1. Origin Version 2021b. OriginLab Corporation. Northampton, MA, USA.
